# CD4^+^ T Cell Differentiation in Infection: Amendments to the Th1/Th2 Axiom

**DOI:** 10.3389/fimmu.2015.00198

**Published:** 2015-04-29

**Authors:** Dragana Jankovic, Carl G. Feng

**Affiliations:** ^1^Laboratory of Parasitic Diseases, National Institute of Allergy and Infectious Diseases (NIAID), National Institutes of Health (NIH), Bethesda, MD, USA; ^2^Department of Infectious Diseases and Immunology, Sydney Medical School, The University of Sydney, Sydney, NSW, Australia

**Keywords:** Th lymphocytes, cytokines, infection, dendritic cells, macrophages, immunoregulation, metabolism, memory, Inc-RNA

CD4^+^ T helper (Th) lymphocytes play a central role in orchestrating immune responses. While the specificity of naïve CD4^+^ T cells is fixed and constrained by the TCR they express, their effector potential is flexible and unbiased. After antigen encounter, Th lymphocytes acquire a specific effector function by responding to the summation of input signals provided by antigen-presenting cells (APC) and cytokine microenvironment. The functional diversity of Th cells provides the immune system with the capacity to mount an appropriate defense mechanism against various types of pathogens.

The initial discovery of the existence of specialized Th effector populations came from an analysis of mouse CD4^+^ T cell clones by Mosmann and Coffman (1). This seminal study demonstrated that differentiated CD4^+^ T cells can be classified into two groups, designated Th1 and Th2 cells, based on their cytokine production. Th1 lymphocytes, which are defined by secretion of IFN-γ, TNF, and IL-2, promote cell-mediated immunity and control infections with intracellular pathogens. In contrast, Th2 lymphocytes, which produce IL-4, IL-5, IL-10, and IL-13, mediate humoral immune responses and resistance to helminth parasites. In addition, the Th1/Th2 dichotomy was also demonstrated in immunopathological settings where Th1 and Th2 cells are implicated in autoimmune diseases and allergic conditions, respectively.

The recognition that different CD4^+^ T cell subsets are associated with specific outcomes in both infection diseases and immune disorders propelled research into the Th1/2 paradigm. Generation of mature Th effectors was defined as an endpoint of a multistep lineage-specific differentiation process in which naïve CD4 T lymphocytes gain the ability to produce exclusively Th1 or Th2 cytokines. Moreover, a similar concept of dichotomous Th1/2 immune functions has been put forward for other lymphocyte populations (Tc1/Tc2), as well as other types of immune cells such as macrophages (M1/M2) and dendritic cells (DC1/DC2).

However, significant technical advances in CD4^+^ T cell biology research over past 20 years have revealed that the Th1/Th2 paradigm cannot fully explain the complexity of Th effectors and led to the discovery of new Th subsets that have distinct yet overlapping functions with Th1/Th2 cells (2–4). For example, Th17 cells, which produce IL-17, are important in controlling extracellular bacterial and fungal pathogens, but can also promote autoimmune disorders (5, 6). Similarly, Tfh cells, which produce IL-21, are important for germinal center formation and antibody production, have taken on some of the functions originally attributed to Th2 cells (7, 8). Together, these findings clearly challenge Th1/2 concept and the model of Th effector choice as a bidirectional and linear differentiation process. Indeed, new molecular techniques that enable comparative analysis between genome-wide landscape of different transcriptional factors and cell-specific transcriptional output revealed that Th polarization is a flexible course of progressions that allows different degrees of functional specialization and diversity among Th cells.

The Research Topic presented here, “CD4 T cell differentiation in infection: an amendment to Th1/Th2 axiom” is a collection of reviews that cover the most recent progress on Th effector choice mechanisms in various infection models.

The Topic opens with two reviews on the functional dichotomy of innate immune cells. The review by Muraille et al. (9) focuses on classically (M1) and alternatively (M2) activated macrophages and their distinct metabolic programs that can be exploited by pathogens as immune evasion strategies. The paper by Hussaarts et al. (10) describes the mechanisms by which helminth components condition dendritic cells for Th2 differentiation and discusses Th2-associated inflammatory responses in the context of metabolic disorders. The immune response to intestinal worm infections is also the topic of the review by Bouchery et al. (11), which focuses on Th2-polarizing signals in CD4^+^ T lymphocytes and the relative contribution of Th2 vs. recently discovered new Th subsets to helminth immunity. The CD4^+^ T cells responses during fungal and malaria infection originally characterized based on Th1/Th2 paradigm are re-examined by Borghi et al. (12) and Perez-Maliah and Langhorne (13), respectively. Borghi et al. (12) describe different Th effectors implicated in anti-fungal resistance and tolerance, while Perez-Maliah and Langhorne (13) discuss the types of Th subsets, including the population of self-controlling multifunctional IL-10^+^ Th1 cells, induced during malaria infection. The review by Engwerda et al. (14) focuses on the mechanisms underlying IL-10 expression in Foxp3^−^ IL-10^+^ Th1 cells and Foxp3^+^ CD4^+^ T regulatory lymphocytes, as well as the role these two populations play in host-protection during protozoan infection.

The notion of CD4^+^ T cell heterogeneity beyond Th1/Th2 effectors is also supported by studies in humans as reviewed by Geginat et al. (15). The molecular mechanisms controlling plasticity vs. stability are just beginning to emerge in both murine and human Th lymphocytes. The paper by Panzeri et al. (16) presents evidence that long intergenic non-coding RNAs (lncRNA) play a role in the Th1 differentiation program of human CD4^+^ T lymphocytes.

Compartmentalized microenvironments with tissue specific conditions (e.g., APC, cytokines) may also contribute to the plasticity of Th effectors. This is certainly true for Th differentiation in gut-associated lymphoid tissue, as discussed by Brucklacher-Waldert et al. (17). Ongoing discoveries of the variable but significant degrees of flexibility among lineage committed CD4 T cells cast doubts on “*bona fide*” Th effector memory responses. Although the Tfh effector subset is considered to be highly unstable, the review by Hale and Ahmed (18) upholds the concept that memory Tfh cells are generated during a viral infection.

The Th1/Th2 paradigm has revolutionized the concept of CD4^+^ T cell differentiation. However, recent discoveries of additional T cell subsets have revealed previously unexpected complexity in the CD4^+^ effector T cell decision-making process. Together, the papers presented here review our current understanding of Th effector choice in infection and emphasize the importance of defining molecular pathways dictating specificity vs. diversity and stability vs. plasticity in CD4^+^ T cells. Ultimately, these new advances will have important implications for rational design of better vaccines and immunotherapies.

## Conflict of Interest Statement

The authors declare that the research was conducted in the absence of any commercial or financial relationships that could be construed as a potential conflict of interest.
